# The E3 ubiquitin ligase, FBXW5, promotes the migration and invasion of gastric cancer through the dysregulation of the Hippo pathway

**DOI:** 10.1038/s41420-022-00868-y

**Published:** 2022-02-24

**Authors:** Yangyang Yao, Zhen Liu, Shanshan Huang, Chunye Huang, Yuan Cao, Li Li, Hui Guo, Fenfen Liu, Shipeng Huang, Quan Liao, Xin He, Jun Chen, Junhe Li, Xiaojun Xiang, Jianping Xiong, Jun Deng

**Affiliations:** 1 Department of Oncology, First Affiliated Hospital of Nanchang University, Nanchang, Jiangxi Province 330006 China; 2 Jiangxi Provincial People’s Hospital, Nanchang, Jiangxi Province 330006 China; 3 Department of Emergency medicine, First Affiliated Hospital of Nanchang University, Nanchang, Jiangxi Province 330006 China

**Keywords:** Gastrointestinal cancer, Tumour biomarkers

## Abstract

F-box and WD repeat domain-containing 5 (FBXW5), with WD40 repeats, can bind to the PPxY sequence of the large tumor suppressor kinases 1/2 (LATS1/2) kinase domain, resulting in ubiquitination. Ubiquitination and the subsequent degradation of LATS1/2 abrogate the Hippo pathway and worsen gastric cancer (GC). However, the effects and molecular mechanisms of FBXW5 in GC remain unexplored. To elucidate the clinical significance of FBXW5, immunohistochemistry was conducted to reveal the positive correlation between FBXW5 expression and lymph node metastasis (*p* < 0.001) and TNM stage (training cohort: *p* = 0.018; validation cohort: *p* = 0.001). Further, patients with high FBXW5 expression were found to have poor prognosis (training cohort: log-rank *p* = 0.020; validation cohort: log-rank *p* = 0.025). Cell experiments revealed the promoting effects of FBXW5 on the proliferation, invasion, metastasis, and chemoresistance of GC cells. Blocking LATS1-YAP1 leads to the loss of FBXW5-mediated regulation of the Hippo pathway and partial functions. Further, co-immunoprecipitation and in vivo ubiquitination assays revealed the interaction between FBXW5 and LATS1, which promoted the ubiquitination and degradation of LATS1. Based on mouse xenograft assays, FBXW5 silencing attenuated the growth of subcutaneous tumor xenografts. Altogether, FBXW5 was found to inactivate the Hippo signaling pathway by enhancing LATS1 ubiquitination and degradation, which promoted the invasion, metastasis, and drug resistance of GC cells.

## Introduction

Approximately 670,000 new gastric cancer (GC) cases and 490,000 GC-related deaths are reported each year in China [1]. Patients with GC have a poor five-year survival rate (less than 40%) owing to a lack of early diagnostic indicators and limited treatment avenues [1, 2]. Uncovering the complicated biological mechanisms underlying GC is thus imperative, which will enable clinical diagnosis, treatment, and fresh cogitation.

The Hippo pathway is involved in GC cell malignancy [3–5]. Abrogating the Hippo pathway signal impairs LATS1/2-mediated phosphorylation of yes-associated protein (YAP) or WW domain-containing transcription regulator 1 (TAZ) [6], which acts as a transcriptional coactivator with TEA domain-containing sequence-specific transcription factors after translocation to the nucleus to activate the transcription of downstream target genes, ultimately degrading GC [7]. Based on increasing evidence, ubiquitination and deubiquitination play important roles in the uncontrolled Hippo pathway [8]. Previously, our group revealed that the E3 ubiquitin ligase molecule, Cullin 4 A (CUL4A), acts as an oncogene by promoting LATS1 ubiquitination and degradation [9]. Further, the deubiquitinating ligase, USP9X, was reported to deubiquitinate and stabilize the LATS2 protein [10]. Determining the effects of ubiquitination and deubiquitination on the Hippo pathway and phenotype is of remarkable significance and will facilitate the discovery of novel molecular targets.

FBXW5, a newly identified member of the F-box protein family, was initially found to inactivate interleukin (IL)-1β-induced JNK/p38 MAPKs and nuclear factor (NF)-κB, and the phosphorylation of Thr187 by transforming growth factor-β-activated kinase 1 (TAK1) [11]. Thereafter, FBXW5 was proposed to bind to the centriolar protein, *Homo sapiens* spindle assembly abnormal protein 6 homolog (HsSAS-6), and promote its ubiquitination and degradation, thereby regulating centrosome duplication and affecting cell cycle regulation [12]. As a result, the researchers proposed that the HsSAS-6 protein is a key substrate in the SCF-FBXW5 complex, and FBXW5 may contribute to tumorigenesis [12]. FBXW5 was reported to interact with cullin 4A–RING ubiquitin ligase–DNA damage-binding protein 1 (CUL4A–DDB1) and act as a major substrate recognition component for the ubiquitination and degradation of deleted in liver cancer 1 (DLC1), which promotes tumor progression. However, the effects of FBXW5 on GC progression and patient prognosis remain unclear [13].

Based on our results, a high level of FBXW5 underlies tumor invasion, lymph node metastasis, TNM stage, and poor prognosis in GC patients. FBXW5 inactivated the Hippo signaling pathway by binding to LATS1 and promoting LATS1 ubiquitination and degradation, which in turn promoted the invasion, metastasis, and chemoresistance of GC cells. Overall, the findings of this study provide novel insights into targeted therapy for GC.

## Results

### High FBXW5 expression leads to poor prognosis in patients with GC

**Fig. 1:**
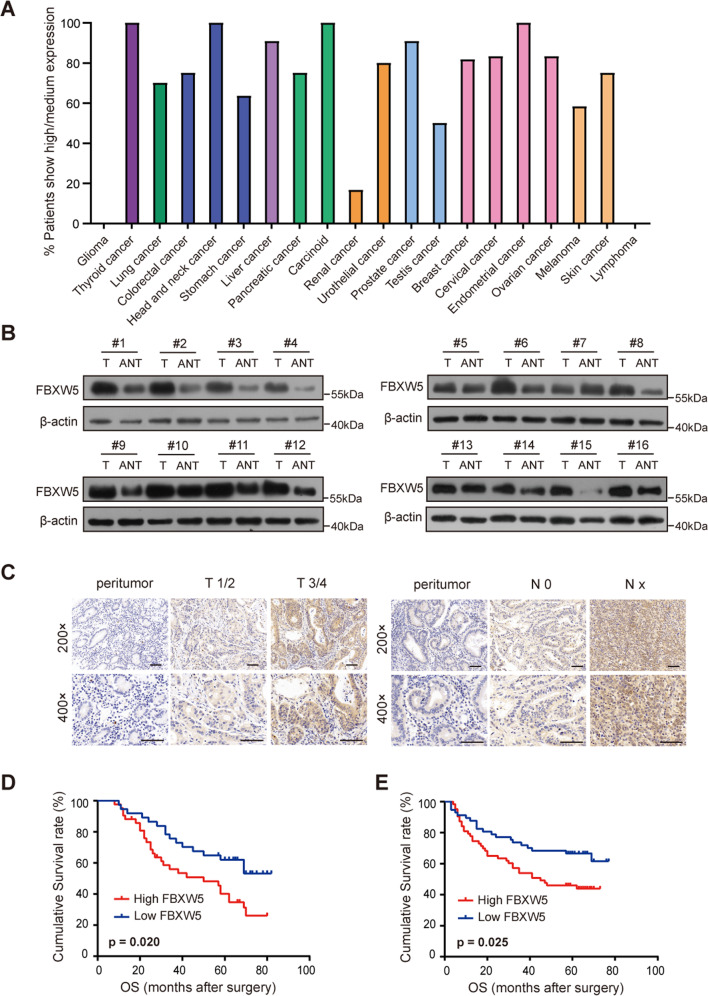
High FBXW5 expression leads to poor prognosis in patients with GC. **A** The protein expression of FBXW5 in various cancers is based on the Human Protein Atlas database. **B** FBXW5 protein level in 16 matched samples of surgically resected gastric tumors and adjacent normal tissues. **C** Representative IHC images of paraffin-embedded gastric tumor tissue specimens. Bar scale: 50 μm. **D** Kaplan–Meier survival curves of 79 paraffin-embedded GC specimens (high FBXW5 level, *n* = 42; low FBXW5 level, *n* = 37; log-rank *p* = 0.020). **E** Kaplan–Meier survival curves of 120 paraffin-embedded GC specimens in the validation group (high FBXW5 level, *n* = 63; low FBXW5 level, *n* = 57; log-rank *p* = 0.025).

We initially searched the Human Protein Atlas Image Classification database (https://www.proteinatlas.org/) to determine the expression of FBXW5 in common human malignant tumors. FBXW5 mRNA was broadly detectable in common human malignant cancers, including GC, colorectal cancer, and lung cancer (Fig. S1A). In addition, by retrieving the immunohistochemistry (IHC) staining dataset from the Human Protein Atlas database, we found that the FBXW5 protein is expressed in most of the common human cancers, except lymphoma and glioma. Approximately 60% of GC samples were found to have moderate to high expression of FBXW5 (Fig. 1A).

The online survival prediction website, Kaplan–Meier Plotter (http://kmplot.com/analysis/index.php?p=service&start=1, Affy ID: 223050_s_at), was used to determine the significance of FBXW5 expression in GC. GC patients with high expression of the FBXW5 mRNA were found to exhibit poorer prognosis (log-rank *p* = 0.0025, HR = 1.42 [1.13–1.79]) than those with low FBXW5 expression (Fig. S1B). To validate the expression and significance of FBXW5 in GC, western blot analysis was carried out using 16 matched samples from surgically resected tissues. Based on the results, the FBXW5 level in gastric tumor tissues was higher than that in adjacent normal tissues (Fig. 1B). A total of 79 paraffin-embedded GC tissue specimens were retrieved for a preliminary study (training cohort) on the relationship between FBXW5 expression and the clinicopathological characteristics and prognosis of GC patients. A high expression of FBXW5 was found to be associated with lymph node metastasis (*p* < 0.001), poor TNM stage (*p* = 0.018), and low differentiation (*p* = 0.042) (Table 1). Further, survival and follow-up data of the 79 patients revealed that the high FBXW5 cohort had a markedly shorter overall survival (OS) than the low FBXW5 cohort (log-rank *p* = 0.02) (Fig. 1D). A total of 120 paraffin-embedded GC tissue specimens (validation cohort) were also collected for further analysis. Consistent regression analysis and survival analysis results were found between the validation cohort and the training cohort (Table 1 and Fig. 1D, E). Moreover, the IHC assays revealed that FBXW5 was predominantly observed in the cytoplasm (Fig. 1C).

**Table 1:** Clinical characteristics of patients according to FBXW5 in the training and validation cohorts.

Variables	Training cohort (*n* = 79)				Validation cohort (*n* = 120)			
	High FBXW5	Low FBXW5	*N*	*p* value	High FBXW5	Low FBXW5	*N*	*p* value
Gender								
Male	21	25	46	0.114	42	44	86	0.201
Female	21	12	33		21	13	34	
Age(years)								
<58	26	15	41	0.058	31	29	60	0.855
≥58	16	22	38		32	28	60	
Tumor size (cm)								
<4	22	25	47	0.17	24	38	62	**0.002**
≥4	20	12	32		39	19	58	
Depth of invasion								
T1-T2	16	19	35	0.237	13	25	38	**0.006**
T3-T4	26	18	44		50	32	82	
Lymph node status								
N0	7	22	29	**<0.001**	14	34	48	**<0.001**
N1-N3	35	15	50		49	23	72	
TNM stage								
I/II	16	24	40	**0.018**	20	35	55	**0.001**
III	26	13	39		43	22	65	
Differentiation status								
Poor and undifferentiated	22	11	33	**0.042**	27	26	53	0.761
Well + Moderate	20	26	46		36	31	67	

Bold values indicate statistically significant differences. Chi-squared tests were performed to assess the association between FBXW5 expression and clinicopathological characteristics.

### FBXW5 promotes the invasion, migration, and EMT of GC cells

**Fig. 2:**
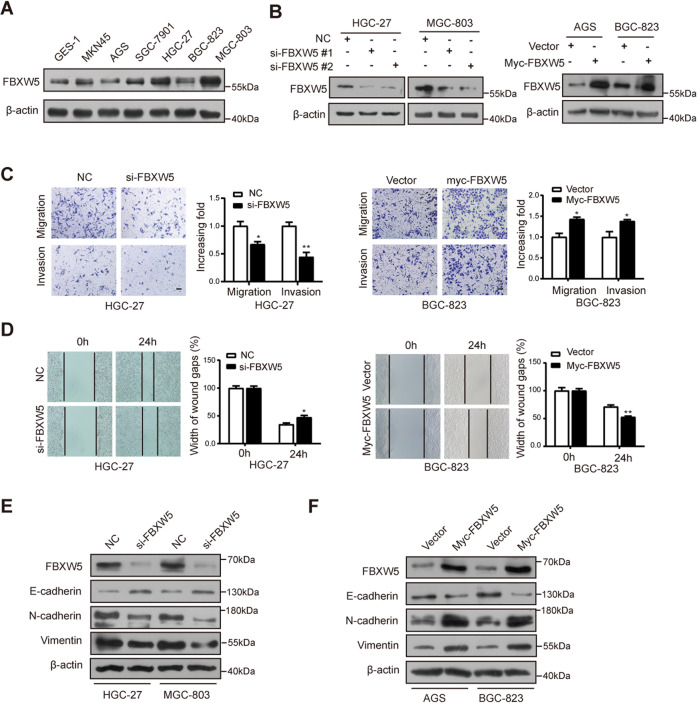
FBXW5 promotes the invasion, migration, and EMT of GC cells. **A** Analysis of FBXW5 level in the GC cell lines. **B** Verification of the transfection efficiency. **C**, **D** After the upregulation or silencing of FBXW5 in GC cells, transwell and wound healing assays were conducted. **E**, **F** Effects of the upregulation or silencing of FBXW5 on the EMT protein. *P < 0.05, **P < 0.01. Bar scale: 100 μm. Data were presented as mean ± SEM from three biologically independent experiments.

FBXW5 was found to be expressed in multiple GC cell lines and was commonly higher in GC cells than in the GES-1 cell (Fig. 2A). FBXW5 was silenced using si-FBXW5 in HGC-27 and MGC-803 cells and overexpressed using the Myc-FBXW5 plasmid in AGS and BGC-823 cells (Fig. 2B). The results of the transwell assays suggested that GC cell migration and invasion abilities were damaged when the expression of FBXW5 was abolished; however, these abilities were strengthened when FBXW5 was overexpressed (Figs. 2C and S2A). Similar results were obtained in the wound healing assays (Fig. 2D). By assessing the EMT protein markers by western blotting, we found that interfering with FBXW5 expression reduced the protein levels of N-cadherin and vimentin, and increased that of E-cadherin (Fig. 2E). In contrast, elevating FBXW5 increased the protein levels of N-cadherin and vimentin, and impaired that of E-cadherin (Fig. 2F). These findings indicate that FBXW5 stimulated GC cell migration, invasion, and EMT.

### FBXW5 promotes chemoresistance in GC cells

**Fig. 3:**
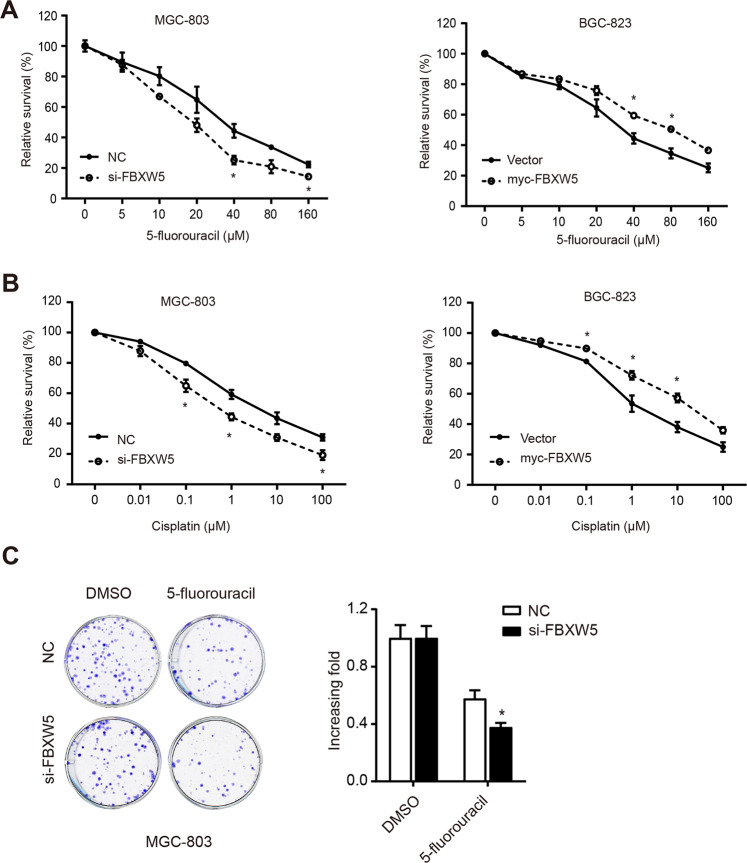
FBXW5 promotes drug resistance in GC cells. **A**, **B** 5-Fluorouracil (5-FU) or cisplatin was added to the transfected cells for the chemo-sensitivity experiments. Thereafter, cellular viabilities were determined using CCK8 analysis. **C** Treatment of transfected MGC-803 with 40 μM 5-FU; colony formation assays were carried out. *P < 0.05, **P < 0.01. The experiments were repeated three times with the most significant results presented.

We conducted CCK8 assays using gradient concentrations of 5-fluorouracil (0, 5, 10, 20, 40, 80, and 160 μM). The downregulation of FBXW5 increased the sensitivity of GC cells to 5-fluorouracil, whereas the upregulation of FBXW5 increased the resistance of cells to 5-fluorouracil (Fig. 3A). Similar results to those obtained for 5-fluorouracil were found using cisplatin (Fig. 3B). Clonogenic assays were performed to demonstrate the increased sensitivity of GC cells to 5-fluorouracil owing to the downregulation of FBXW5. The addition of 5-fluorouracil to MGC-803 cells resulted in a 62% decrease in the number of colonies after the silencing of FBXW5 expression (si-FBXW5 group). However, the NC group only displayed a 42% decrease (Fig. 3C).

### FBXW5 regulates the Hippo signaling pathway

**Fig. 4:**
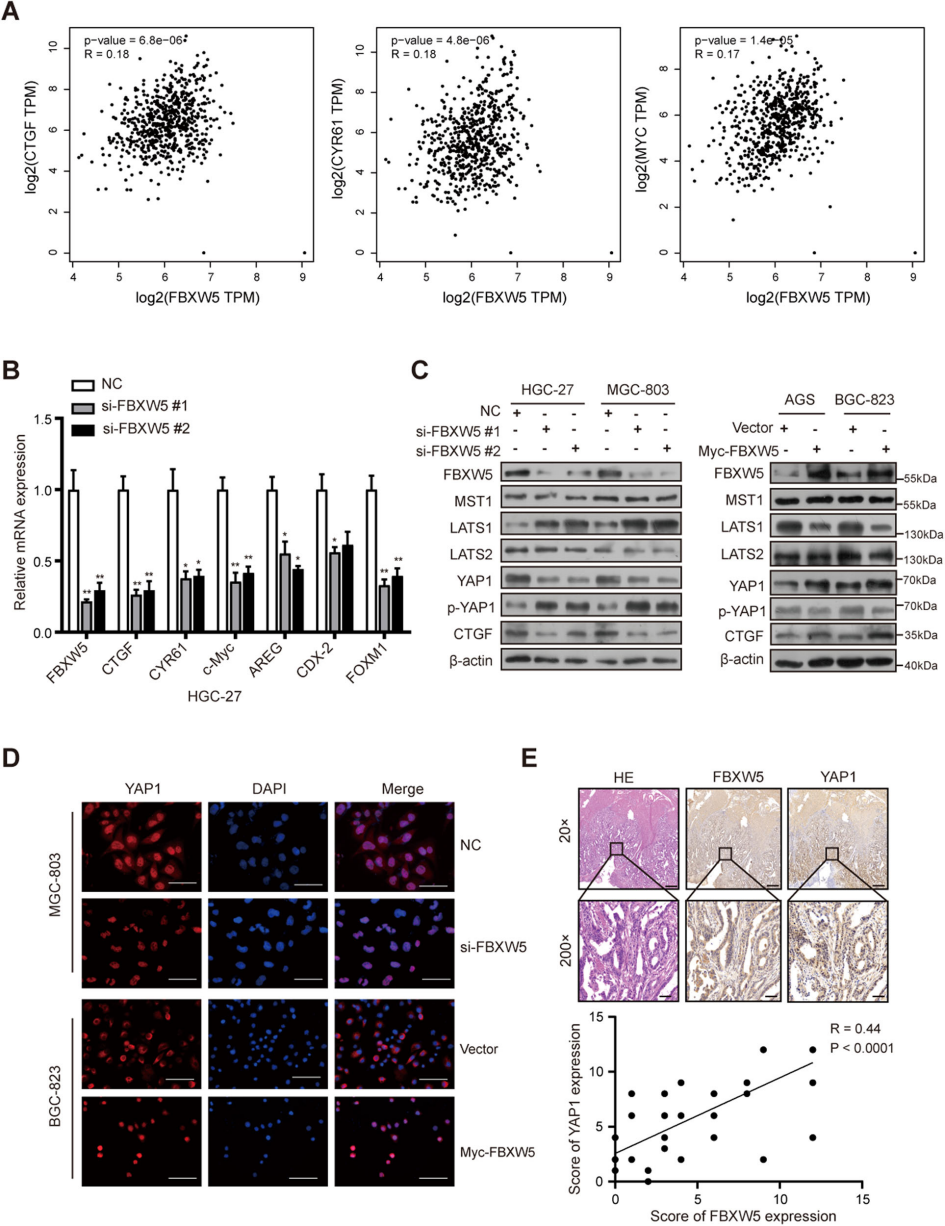
FBXW5 regulates the Hippo signaling pathway. **A** The association between the expression of *FBXW5* and *CTGF*, *CYR61*, and *c-Myc* is plotted on the GEPIA website. **B** Quantitative real-time PCR was performed using the HGC-27 cell line to determine the effect of FBXW5 knockdown on the Hippo pathway target genes at the mRNA level. Data were presented as mean ± SEM from three biologically independent experiments. **C** Western blots were used to determine the effect of FBXW5 knockdown on the Hippo pathway protein. **D** Immunofluorescence results for the YAP1 protein after the knockdown or upregulation of FBXW5 in GC cells. Bar scale: 50 μm. **E** The association between YAP1 and FBXW5 was evaluated via IHC using 45 paraffin-embedded GC samples. Bar scale: 500 μm (20×), 50 μm (200×).

By mining the Cancer Genome Atlas (TCGA) and the Genotype-Tissue Expression (GTEx) databases with the webserver, GEPIA, FBXW5 was found to be positively correlated with the Hippo pathway target genes *CTGF, CYR61*, and *c-Myc* (Fig. 4A). Further, the downregulation of FBXW5 was found to markedly decrease the mRNA expression of *CTGF, CYR61, c-Myc, AREG, CDX-2*, and *FOXM1* (Figs. 4B and S2B). Based on western blotting, the knockdown of FBXW5 increased the protein levels of LATS1 and p-YAP1 and decreased those of YAP1 and CTGF (Fig. 4C). In contrast, upregulating FBXW5 inhibited LATS1 and p-YAP1 expression and increased YAP1 and CTGF expression (Fig. 4C). However, alterations in the protein levels of MST1 and LATS2 were not observed (Fig. 4C). Immunofluorescence assay revealed that FBXW5 depletion reduced the expression and nuclear translocation of YAP1. In contrast, the upregulation of FBXW5 increased the proportion of YAP1 protein that entered the nucleus (Fig. 4D).

To delineate the relationship between the FBXW5 protein and the Hippo pathway using clinical samples, IHC staining of FBXW5 and YAP1 in 45 paraffin-embedded GC samples was performed. Correlation analysis revealed a positive correlation between FBXW5 and YAP1(*R* = 0.44, *p* < 0.001) (Fig. 4E). Collectively, these results indicate that FBXW5 may regulate the Hippo signaling pathway and promote the nuclear translocation of the YAP1 protein.

### FBXW5 binds to LATS1 and promotes its degradation

**Fig. 5:**
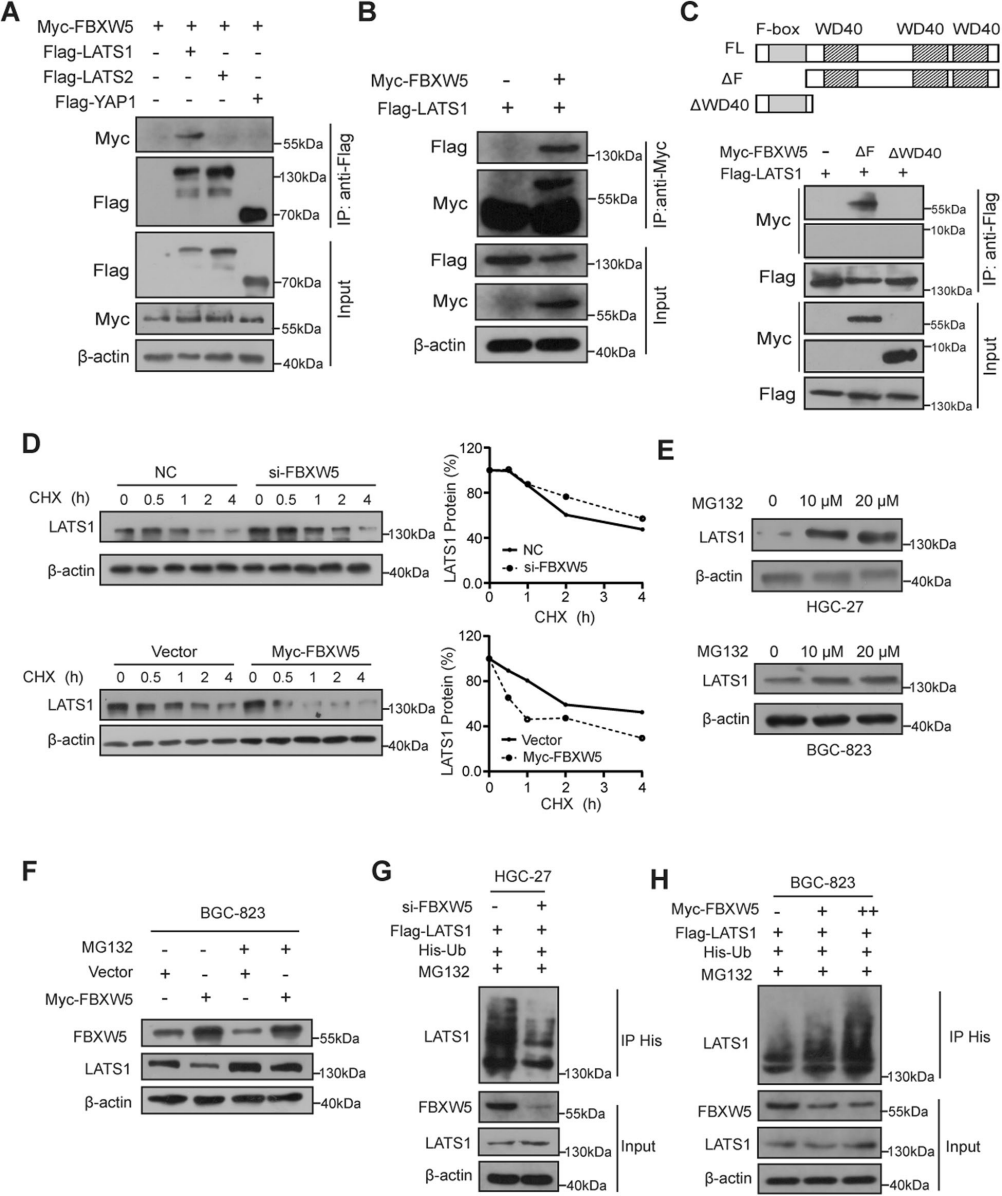
FBXW5 binds to LATS1 and promotes its degradation. **A**, **B** Co-immunoprecipitation assays were carried out using HEK-293T cells after transfection with myc-FBXW5, flag-LATS1, flag-LATS2, or flag-YAP1 plasmid. **C** Co-immunoprecipitation assays were carried out using HEK-293T cells after transfection with myc-FBXW5ΔF-box (ΔF), ΔWD, and flag-LATS1. **D** After cells were treated with 20 μg/ml cycloheximide (CHX) for distinct lengths of time, western blot analysis was conducted. **E** Cells were treated with the proteasome inhibitor, MG132, and the protein level of LATS1 was determined using western blot analysis. **F** BGC-823 cells overexpressing FBXW5 were treated with MG132 for 6 h and analyzed to determine LATS1 expression. **G**, **H** In vivo protein ubiquitination assays were carried out using the GC cell lines, HGC-27 and BGC-823.

Changes in the level of FBXW5 could lead to changes in the levels of the LATS1 and YAP1 proteins. To clarify the specific mechanism by which FBXW5 regulates the Hippo pathway, we performed co-immunoprecipitation (Co-IP) assays using HEK-293T cells and confirmed the binding of exogenous LATS1 protein with exogenous FBXW5 protein (Fig. 5A, B). FBXW5 is composed of two recognized domains: an N-terminally located F-box motif and seven WD40 repeats occupying most of the rest of the sequence [14]. To identify the specific interaction domain, deletion mutants of the F-box motif and WD40 domain of FBXW5 (ΔF-box and ΔWD) were used for a co-immunoprecipitation assay, ΔF-box but not ΔWD FBXW5 coimmunoprecipitated with LATS1, suggesting that WD40 repeats of FBXW5 were required for interaction with LATS1 (Fig. 5C). Thereafter, we used CHX to inhibit protein synthesis in GC cells and found that the downregulation of FBXW5 reduced the rate of LATS1 degradation and prolonged its half-life. In contrast, the upregulation of FBXW5 accelerated LATS1 protein degradation and shortened its half-life (Fig. 5D). To explore the mechanism by which FBXW5 regulates LATS1 protein expression, we first confirmed that blocking proteasomes curbed LATS1 degradation, which indicates that LATS1 was markedly degraded via the proteasome pathway (Fig. 5E). Furthermore, after blocking the proteasome pathway with MG132, the effect of FBXW5 on LATS1 disappeared (Fig. 5F), demonstrating that FBXW5 acts on LATS1 through the ubiquitin-proteasome pathway. We proceeded to conduct in vivo protein ubiquitination assays using HGC-27 and BGC-823 cells. Based on our results, the silencing of FBXW5 expression in GC cells attenuated the ubiquitination of LATS1, whereas the upregulation of FBXW5 promoted LATS1 ubiquitination (Fig. 5G, H). Ubiquitination levels were also found to increase with the degree of FBXW5 upregulation (Fig. 5H). Such results indicate that FBXW5 regulates the Hippo signaling pathway by binding and promoting LATS1 ubiquitination and degradation.

### Silencing of FBXW5 attenuates the growth of tumor xenografts

**Fig. 6:**
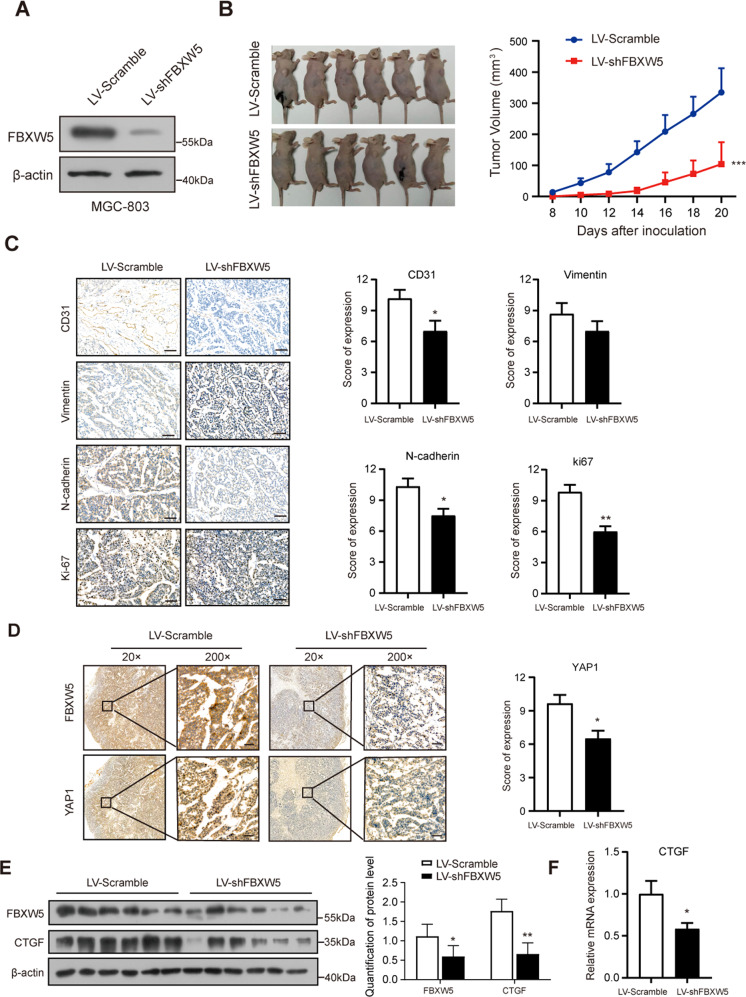
Silencing of FBXW5 attenuates the growth of tumor xenografts. **A** Western blot verification of the efficiencies of lentivirus transfection. **B** Xenograft tumors were harvested after monitoring for 20 days, and growth curves were plotted using xenograft tumors derived from the LV-scramble-shRNA group or LV-shFBXW5 group (mean ± SD, *n* = 6). **C** IHC was carried out and the results were quantified using the grading standards described above (mean ± SEM, *n* = 6). **D** IHC was conducted to determine the expression of FBXW5 and YAP1, and the results were quantified using the grading standards described above (mean ± SEM, *n* = 6). **E** Western blotting was used to detect the FBXW5 and CTGF proteins in six xenograft tumors. The protein levels of FBXW5 and CTGF were quantified and normalized to the level of β-actin. **F** The mRNA levels of *CTGF* were detected in six xenograft tumors using qPCR (mean ± SEM, *n* = 6). *P < 0.05, **P < 0.01, ***P < 0.001.

We validated the function of the FBXW5 protein in vivo by constructing a subcutaneous xenograft tumor model in nude mice. We transfected MGC-803 cells with lentivirus harboring shFBXW5 or scramble and generated a stable cell line through antibiotic selection. The silencing of FBXW5 was validated by western blotting, and subcutaneous inoculation was subsequently performed (Fig. 6A). The tumor growth curve indicated that interfering with FBXW5 expression profoundly impaired xenograft tumor growth rate (Fig. 6B). Further, IHC staining of the xenograft tumor tissue revealed that CD31, vimentin, N-cadherin, and Ki-67 were repressed when FBXW5 was depleted. Such alterations in CD31, N-cadherin, and Ki-67 were found to be statistically significant (Fig. 6C). IHC also revealed that the YAP1 protein was significantly reduced in the LV-shFBXW5 group, and the expression of YAP1 in the nucleus was decreased (Fig. 6D). The control group had higher FBXW5 expression than the LV-shFBXW5 group. The expression of YAP1 was also higher and more clusters were visible in the nucleus (Fig. 6D). Based on subsequent experiments, the protein and mRNA levels of CTGF were significantly suppressed in the LV-shFBXW5 group (Fig. 6E, F). Collectively, the silencing of FBXW5 may reduce the entry of YAP1 into the nucleus and inhibit the growth of tumor xenografts.

### Blocking LATS1-YAP1 leads to the loss of FBXW5-mediated regulation of the Hippo pathway and partial functions

**Fig. 7:**
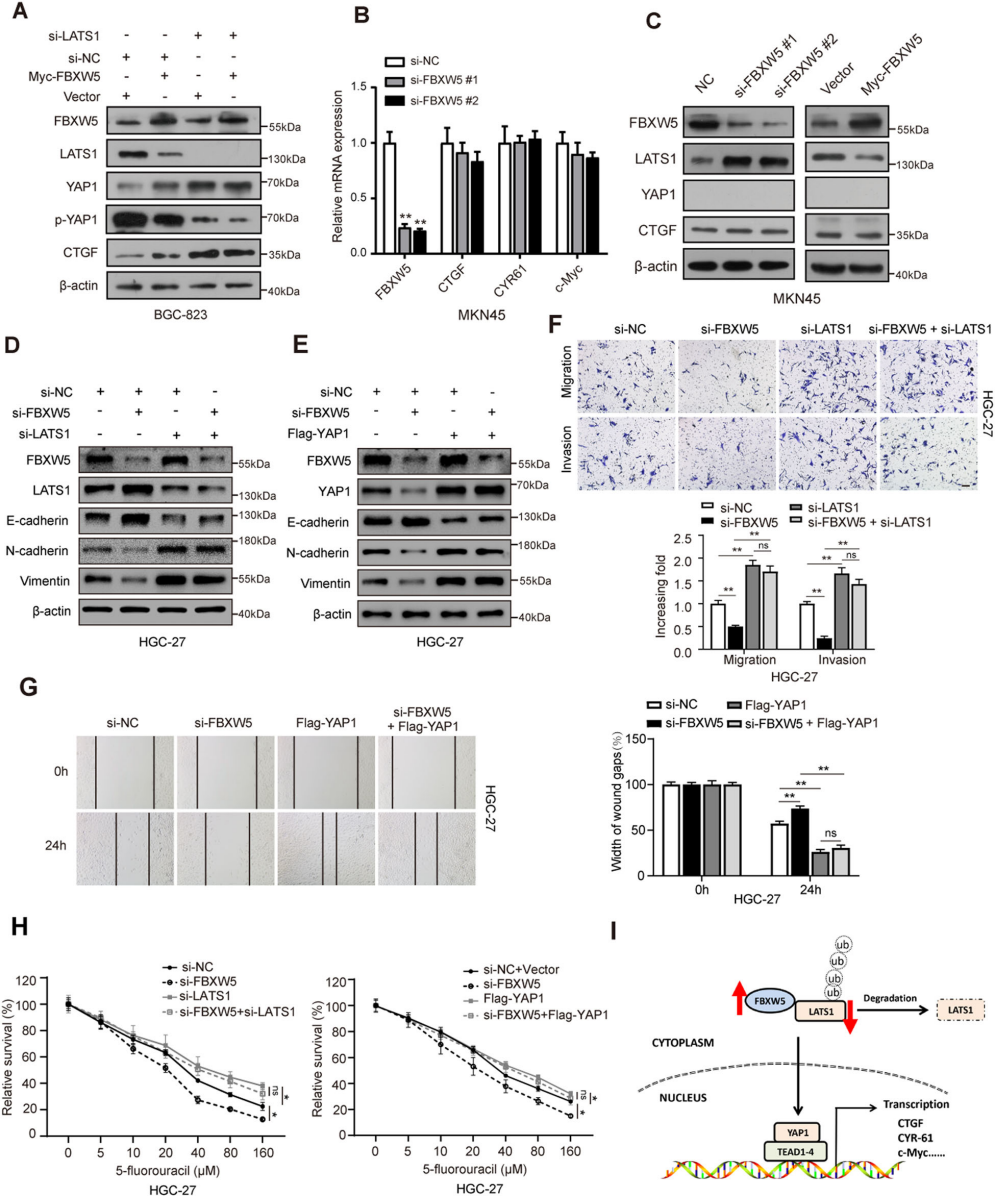
Blocking LATS1-YAP1 leads to the loss of FBXW5-mediated regulation of the Hippo pathway and partial functions. **A** Silencing of LATS1 and upregulation of FBXW5 in BGC-823 cells. Western blot analysis was conducted to verify the effect of FBXW5 on the Hippo pathway during LATS1 deficiency. **B** RT-qPCR was carried out to determine the effect of FBXW5 knockdown on the downstream molecule of the Hippo pathway in MKN-45 cells. Data were presented as mean ± SEM from three biologically independent experiments. **C** Western blotting was performed using MKN-45 to determine the effect of FBXW5 on protein expression in the Hippo pathway. **D**, **E** Effects of FBXW5 silencing on the EMT protein after LATS1 knockdown or YAP1 supplementation. **F**, **G** After LATS1 knockdown or YAP1 supplementation, transwell and wound healing assays were conducted to determine the effect of FBXW5 on cell invasion and migration. The experiments were repeated three times with the most significant results presented. **H** After LATS1 knockdown or YAP1 overexpression, 5-fluorouracil (5-FU) was added to the transfected cells for the chemo-sensitivity experiments. Thereafter, cell viabilities were determined using the CCK8 analysis. The experiments were repeated three times with the most significant results presented. **I** Schematic of FBXW5 participating in GC. Bar scale: 100 μm.

To determine whether the mechanism underlying FBXW5 function is mediated by the LATS1-YAP1 axis, we silenced LATS1 in BGC-823 cells using siRNA. After LATS1 silencing, FBXW5 could not enhance the levels of YAP1 and its downstream molecule, CTGF (Fig. 7A), indicating the regulation of YAP1 and its downstream target genes by FBXW5 is mediated via LATS1. Of the six GC cell lines, the MKN-45 cell line showed a deficiency in YAP1 protein expression due to naturally occurring homozygous loss-of-function variants [15] (Fig. S3). Further, qPCR revealed that the mRNA expression of the Hippo pathway downstream target genes did not change with alterations in FBXW5 levels in MKN-45 cells (Fig. 7B). Western blotting showed that although the downregulation or upregulation of FBXW5 could increase or decrease LATS1 protein levels, the regulatory effect of FBXW5 on the downstream target, CTGF, was lost because of the deficiency of the YAP1 protein (Fig. 7C).

To determine whether the LATS1-YAP1-Hippo pathway makes an essential contribution to FBXW5-mediated functions, such as the regulation of EMT, malignant properties, and chemoresistance of GC cells, we knocked down LATS1 and FBXW5 in HGC-27 cells. When LATS1 was knocked down, the protein levels of N-cadherin, vimentin, and E-cadherin did not significantly change after FBXW5 silencing (Fig. 7D). The exogenous transfer of YAP1 can eliminate the regulatory effect of FBXW5 silencing on the EMT protein (Fig. 7E). Transwell and wound healing assays revealed that the invasion and migration ability of GC cells will be significantly affected by changes in FBXW5. However, knocking down LATS1 or upregulating YAP1 will eliminate the weakening effect of the invasion and migration ability induced by FBXW5 silencing to a certain extent (Fig. 7F, G). CCK8 assays were subsequently conducted to determine the contribution of the LATS1-YAP1 axis to the chemoresistance of GC cells. Briefly, HGC-27 was treated with a gradient concentration of 5-fluorouracil which is described above. Based on the results, YAP1 can rescue the sensitization of chemotherapy caused by FBXW5 downregulation, while LATS1 silencing can abolish the effect of FBXW5 downregulation on chemoresistance (Fig. 7H).

## Discussion

In this study, clinical gastric tumor samples, cell-based experiments, and nude mouse models were employed to provide the first evidence that FBXW5 binds to and degrades LATS1 through the ubiquitin-proteasome pathway, ultimately inactivating the Hippo signaling pathway (Fig. 7I). These activities promote GC cell invasion, metastasis, and the EMT process.

Proteins in the F-box protein family constitute a subunit of the SCF ubiquitin ligase complex and are crucial for phosphorylation-dependent ubiquitination [16]. The F-box protein family participates in massed physiological processes, including cell cycle regulation, apoptosis, signal transduction, DNA repair, and tumorigenesis [17–20]. By searching the Human Protein Atlas Image Classification database, we found that FBXW5 is broadly expressed in human cancers, including GC. In addition, the Kaplan–Meier Plotter predicted that a high expression of the FBXW5 mRNA was associated with poor prognosis in GC patients. As a result, we speculated that FBXW5, a member of the F-box protein family, may act as a proto-oncogene in GC. Herein, the protein expression of FBXW5 was significantly increased in GC tissue. The protein level of FBXW5 affects lymph node metastasis, depth of invasion, and TNM stage. Therefore, high levels of FBXW5 may induce the development and metastasis of GC. Based on a follow-up analysis of data from two groups of patients (discovery cohort *n* = 79; validation cohort *n* = 120), patients with high FBXW5 protein expression were found to have a poorer prognosis than those with low FBXW5 protein expression.

Failure to cure GC is primarily due to metastasis and chemoresistance. Our transwell and scratch assays revealed that FBXW5 contributes to GC cell invasion and migration. For EMT, epithelial cells were inclined to possess the mesenchymal cell phenotype. Herein, FBXW5 was found to positively regulate vimentin and N-cadherin expression and negatively regulate E-cadherin expression. Thus, FBXW5 may enhance GC cell invasion and metastasizing abilities by promoting EMT. Previously, FBXW5 was proposed to form a complex with CUL4A in non-small cell lung cancer, which led to the degradation of the tumor suppressor protein, DLC1, a Rho GTPase-activating protein, ultimately promoting cell proliferation [13]. FBXW5 is reported to bind to the PPxY sequence of the LATS1/2 kinase domain and lead to the ubiquitination and degradation of LATS1/2 [21].

To validate our hypothesis that FBXW5 regulates the Hippo pathway, we employed the bioinformatics analysis tool, GEPIA, and searched the databases, TCGA and GTEx. Subsequently, we silenced FBXW5 using a siRNA and confirmed the regulatory relationship between FBXW5 and *CTGF*, *CYR61*, *c-Myc*, and other downstream target genes of the Hippo pathway using qPCR. Co-IP assays revealed binding between FBXW5 and LATS1. However, no obvious binding was observed between FBXW5 and LATS2 (a homolog of LATS1) as well as the downstream effector, YAP1. FBXW5 was found to regulate the Hippo signaling pathway by binding to LATS1 and degrading LATS1 via ubiquitination. N-terminally located F-box motif and WD40 repeats domain were two recognized domains of FBXW family proteins, COOH-terminal WD40-repeat domain is often associated with substrate recognition. By constructing FBXW5 deletion mutants (ΔF-box and ΔWD), this study demonstrated that FBXW5 binds to LATS1 via the WD40 repeats suggesting that LATS1 may be the substrate of FBXW5 [22]. The silencing of LATS1 expression or deficiency in the YAP1 protein mitigated or eliminated the regulatory effects of FBXW5 on the Hippo pathway. These findings indicate that the regulation of *CTGF*, *CYR61*, *c-Myc*, and other oncogenes by FBXW5 was mediated via the LATS1-YAP1 axis. Finally, we delineated the significance of the FBXW5-LATS1-Hippo signaling pathway regulation using nude mouse models.

Recently, Mei Shi Yeo et al. reported that FBXW5 may promote GC tumorigenesis and metastasis by activating the FAK-Src signaling pathway, which indicated that FBXW5 might be a potential therapeutic target [23]. Our study results led to similar conclusions, and the relationship between FBXW5 and chemoresistance was further explored. Mechanistically, we proposed that the Hippo pathway may be another important signaling pathway of FBXW5 for regulating GC progression. Cytoplasmic YAP1/TAZ binds to the 14-3-3 protein and is degraded via the SCF^β-TRCP^ E3 ubiquitin-proteasome pathway [8]. Coincidentally, LATS1/2 is regulated by the ubiquitin-proteasome pathway [9, 10]. Accordingly, we identified FBXW5 as a novel E3 ubiquitin ligase that promotes LATS1 protein degradation. The binding of FBXW5 and LATS1 may rely on the direct binding between the WD40 domain of FBXW5 and the PPxY sequence motif of the LATS1/2 kinase domain. However, this proposal will be confirmed in our future investigations. The Hippo pathway can be harnessed as a target for cancer treatment. Moreover, verteporfin has been reported to inhibit the formation of the YAP-TEAD complex and has been approved by the US FDA for use in animal studies and locally advanced pancreatic cancer. Phase I/II clinical trials of verteporfin are ongoing [24, 25]. To our knowledge, this study is the first to report that the dysregulation of the FBXW5-LATS1-YAP1 signaling pathway promotes the malignant progression of GC. Therefore, the development of FBXW5 small-molecule inhibitors as agents for targeted therapy or novel combination therapy may offer new strategies for the treatment of patients with GC.

## Materials and methods

### Patients and tissue specimens

Fresh patient tissues were retrieved from the General Surgery Department of First Affiliated Hospital of Nanchang University. All sixteen patients signed informed consent document before sample collection. A total of 79 paraffin-embedded patient tissues were collected between January 2010 and December 2010 for the training cohort, while 120 tissues were obtained between January 2011 and December 2012 for the validation cohort. Patients did not receive treatment before surgery. The study was approved by the Ethics Committee of the First Affiliated Hospital of Nanchang University.

### Cell lines and culture

The human immortalized gastric epithelial cell line, GES-1, the GC cell lines (AGS, MKN-45, HGC-27, MGC-803, BGC-823, SGC-7901) and HEK-293T cells were obtained from the Shanghai Institute for Life Science, Chinese Academy of Sciences (Shanghai, China). Cells were cultured in RPMI-1640 (Solarbio, China) containing 10% fetal bovine serum (FBS, HyClone; GE Healthcare) at 37 °C in a 5% CO_2_ humidified incubator [26].

### Immunohistochemistry and Immunofluorescence

The detailed IHC and immunofluorescence assay procedures are provided in our previous paper [9]. The primary antibodies are listed in supplemental Table 1. The specimens were evaluated by two independently qualified pathologists, and the grading patterns were consistent with those of our previous work [9]. Immunofluorescence samples were observed under a fluorescence microscope (OLYMPUS, Japan).

### Cell transfection

The specific sequences of siRNA are listed in Supplemental Table 2. The Myc-FBXW5, Flag-YAP1, Flag-LATS1, Flag-LATS2 and vector plasmids were procured from GenePharma (Shanghai, China). Lipofectamine 2000 (Invitrogen, Waltham, MA, USA) was employed as the transfection reagent according to the manufacturer’s protocol. After 48 h of transfection, cells were harvested and used for further in vivo and in vitro experiments.

### Immunoblotting and Co-IP

Western blotting and Co-IP analysis were conducted as previously described [9]. Mutations were introduced by site-directed mutagenesis [14]. The primary antibodies are listed in Supplemental Table 1.

### RNA extraction and qPCR

The protocols for total RNA extraction, reverse transcription and qPCR are provided in our previous paper [25]. The primer sequences used for qPCR are listed in Supplemental Table 3.

### Migration and invasion assays

Cell invasion and migration capacities were evaluated by transwell and wound healing assays, according to methods described previously [26, 27].

### Cell proliferation and colony formation assays

Cell proliferation and colony formation assays were carried out to assess GC cell chemotherapy resistance. Transfected cells were treated with gradient concentrations of 5-fluorouracil or cisplatin. The detailed experimental procedures for the CCK8 and colony formation assays are described in our previous reports [26, 28].

### In vivo ubiquitination assay

Ubiquitination assays were performed using HEK-293T cells, according to the detailed procedure provided in our published paper [29].

### Mouse xenograft assays

A total of 12 female BALB/C nude mice (4 to 6 weeks old) were randomly divided into two groups (LV-shFBXW5 or LV-scramble) with six mice in each group. The animal protocol was approved by the Institutional Animal Care and Ethics Committee of the First Affiliated Hospital of Nanchang University. Scramble short hairpin RNAs or short hairpin RNAs targeting FBXW5 were subcloned into the lentiviral expression vector, GV248 (Genepharma). MGC-803 cells, lentivirus, and puromycin were used to construct FBXW5 stably silenced cells. The detailed animal experiment procedure is described in our previous report [26].

### Statistical analysis

Data are expressed as mean ± SEM of three independent experiments. The difference between groups was determined using unpaired two-tailed Student’s *t*-test. Chi-squared tests were performed to assess the association between FBXW5 expression and clinicopathological characteristics. Survival curves were plotted by the Kaplan–Meier method using GraphPad Prism V8. SPSS Statistics 23 (Chicago, IL, USA) was used for all statistical analyses. A *P* value <0.05 was considered to indicate statistical significance (*P < 0.05 and **P < 0.01).

## Supplementary information

western blots original data

Supplementary figure and table legends

Supplementary Tables

Supplementary Figure S1

Supplementary Figure S2

Supplementary Figure S3

## Data Availability

The raw data supporting the conclusions of this article will be made available by the authors, without undue reservation.
