# Supplementary material for: The E3 ubiquitin ligase, FBXW5, promotes the migration and invasion of gastric cancer through the dysregulation of the Hippo pathway
Source: Cell Death Discov. 2022 Feb 24;8:79. doi: 10.1038/s41420-022-00868-y (PMC8873275; doi:10.1038/s41420-022-00868-y)

FIGURE 1B

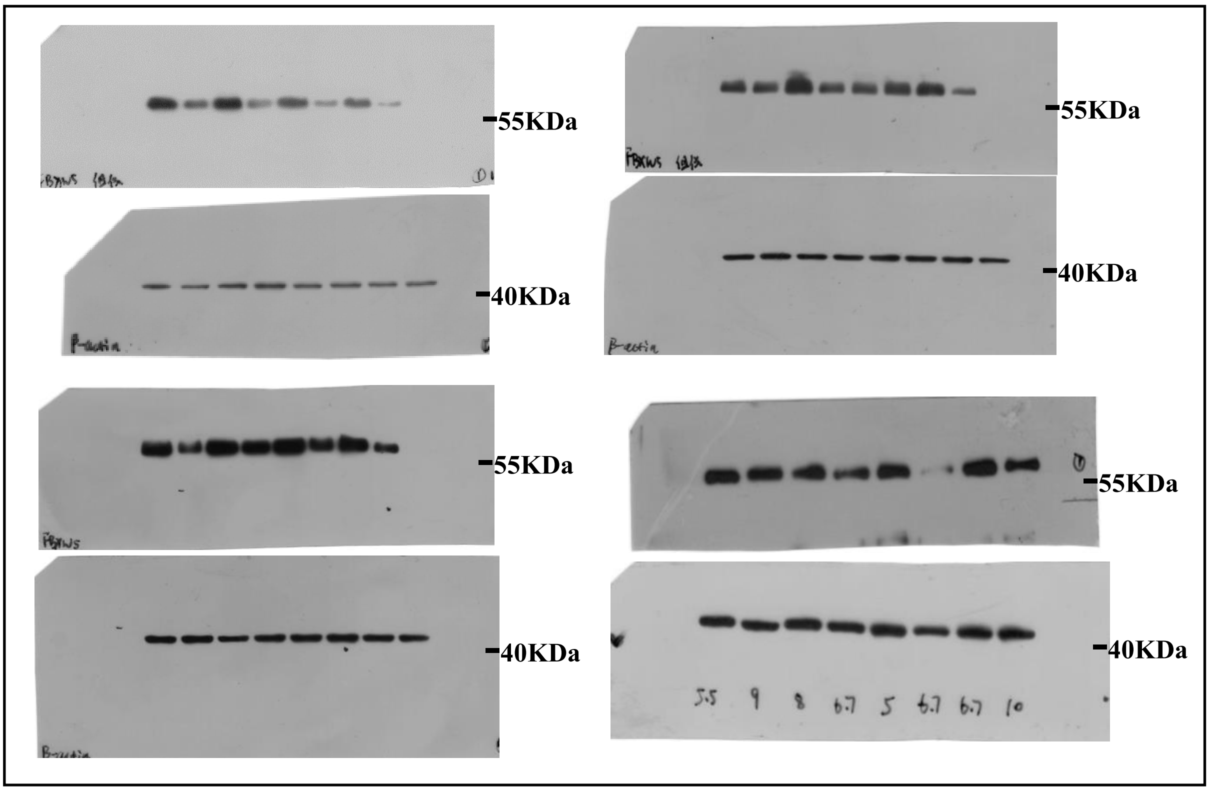

FIGURE 2A-B

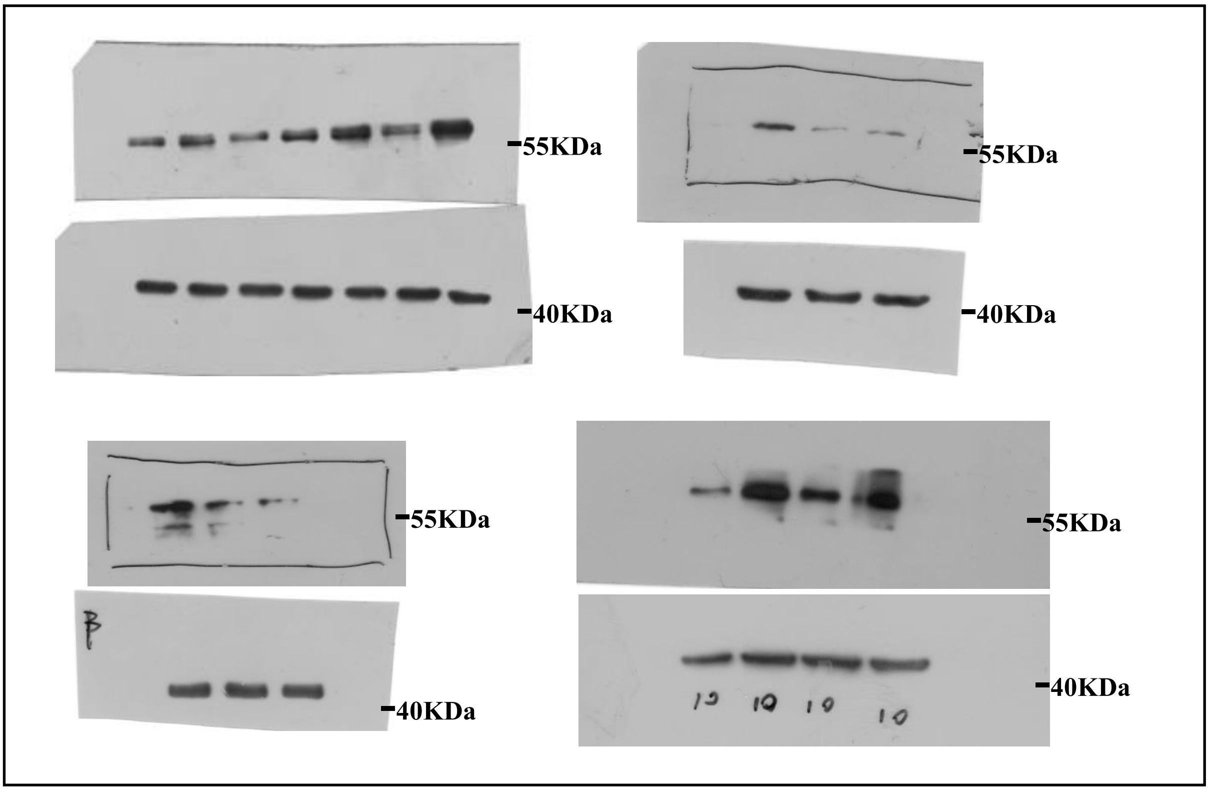

FIGURE 2E-F

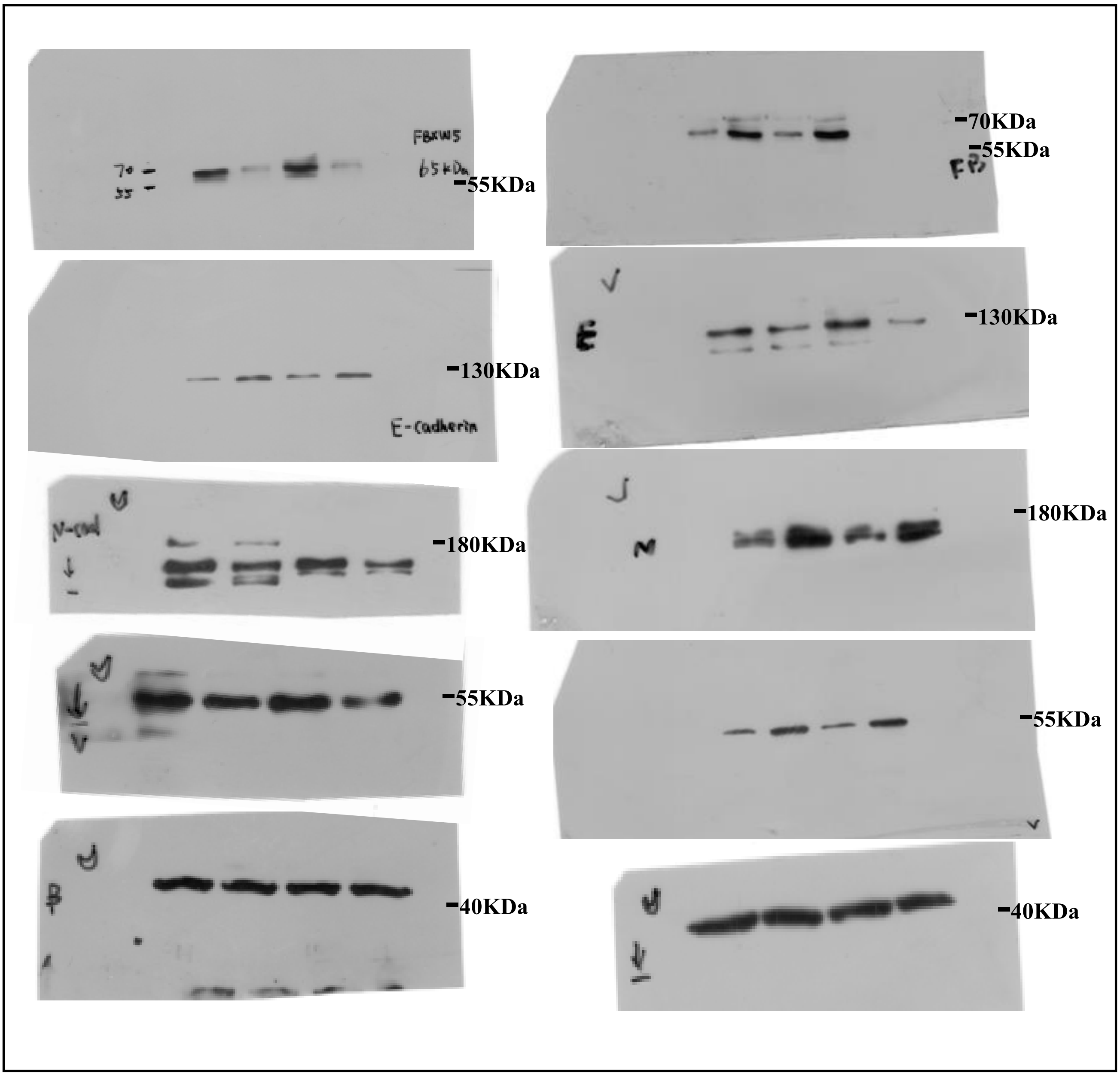
FBXW5
70
55
65KDa
55KDa
70KDa
55KDa
FP
130KDa
E-cadherin
E
130KDa
N-cad
180KDa
N
180KDa
55KDa
55KDa
40KDa
40KDa

FIGURE 4C

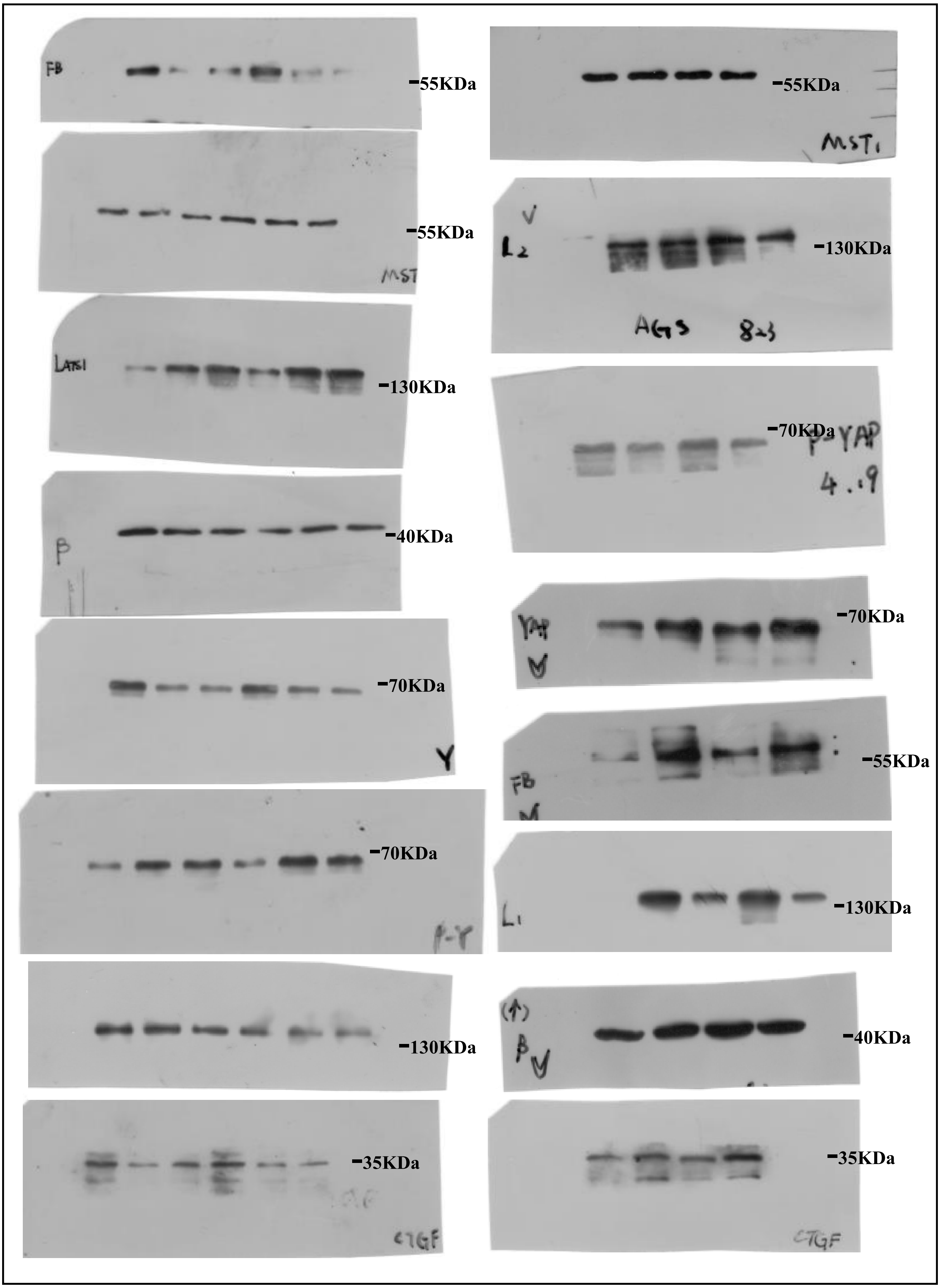
FB
-55KDa
MST
-55KDa
LATS1
-130KDa
β
-40KDa
-70KDa
Y
-70KDa
P-Y
-130KDa
-35KDa
CTGF
-55KDa
MST1
V
L2
-130KDa
AGS 823
-70KDa
P-YAP
4.19
YAP
-70KDa
FB
-55KDa
L1
-130KDa
(↑)
β
-40KDa
-35KDa
CTGF

FIGURE 5A-C

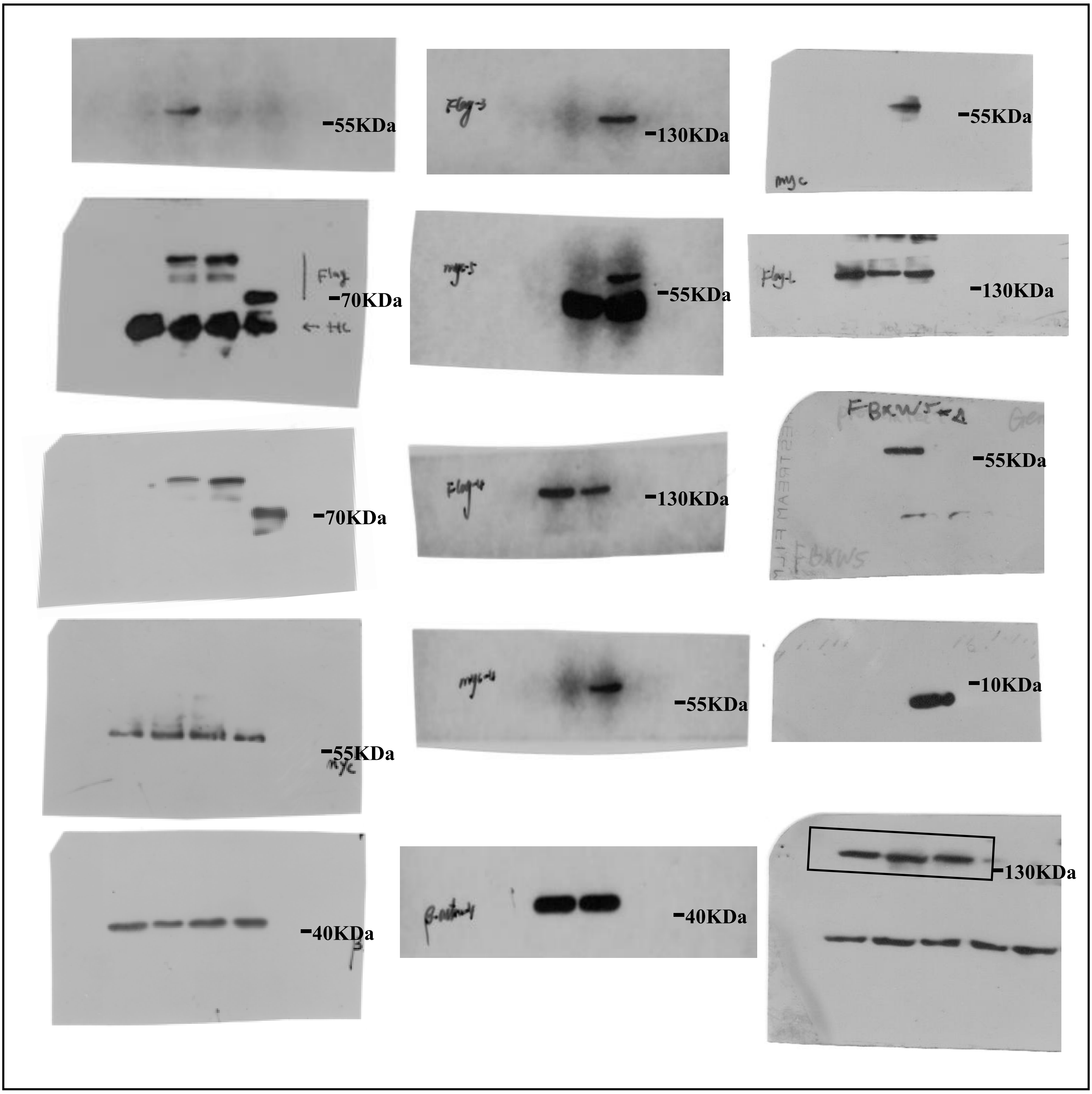
-55KDa
-130KDa
-55KDa
-70KDa
-55KDa
-130KDa
-70KDa
-130KDa
-55KDa
-55KDa
-55KDa
-10KDa
-40KDa
-40KDa
-130KDa

FIGURE 5D-F

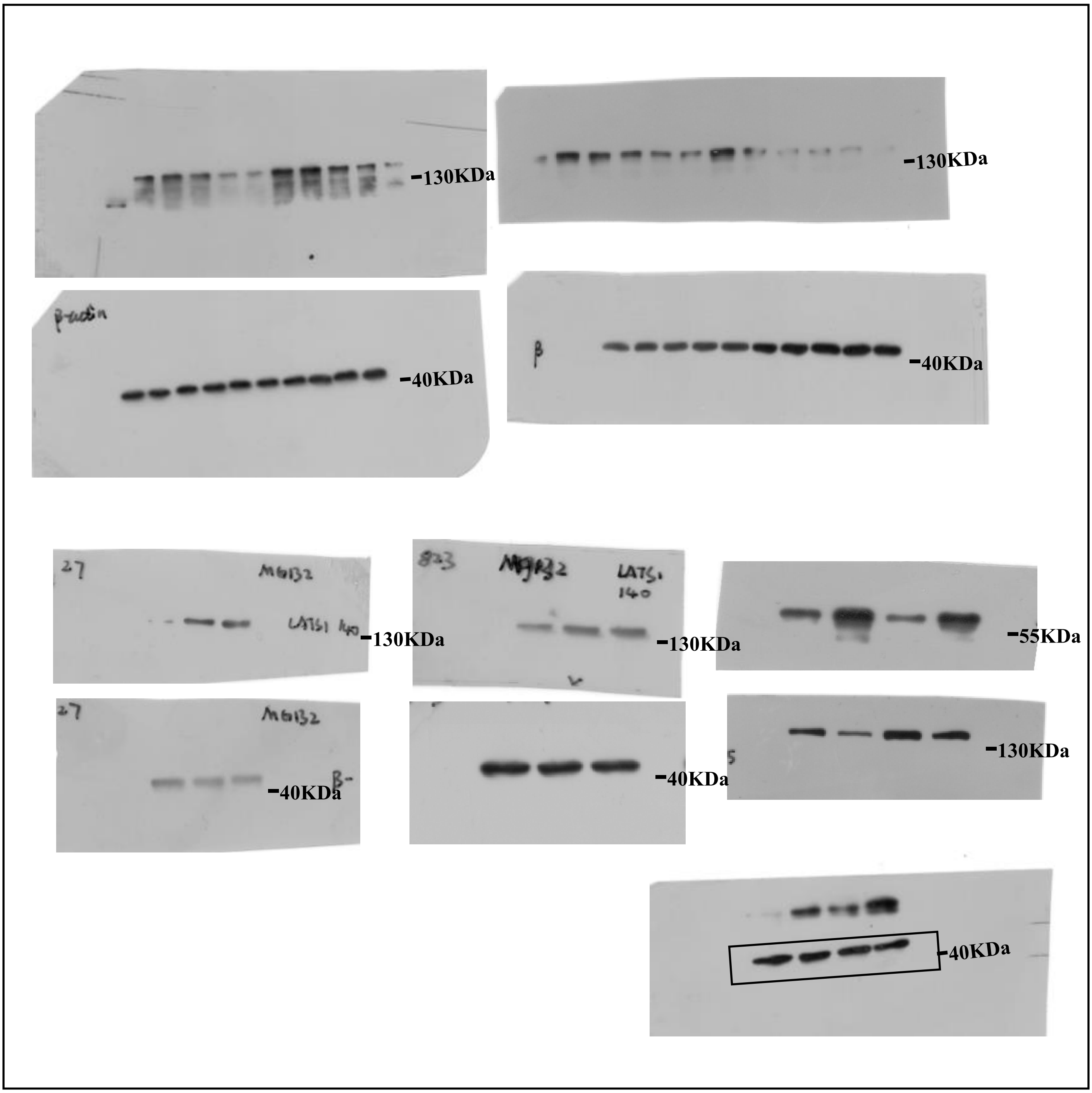
-130KDa
-130KDa
-40KDa
-40KDa
-130KDa
-130KDa
-55KDa
-40KDa
-40KDa
-130KDa
-40KDa

FIGURE 5G-H

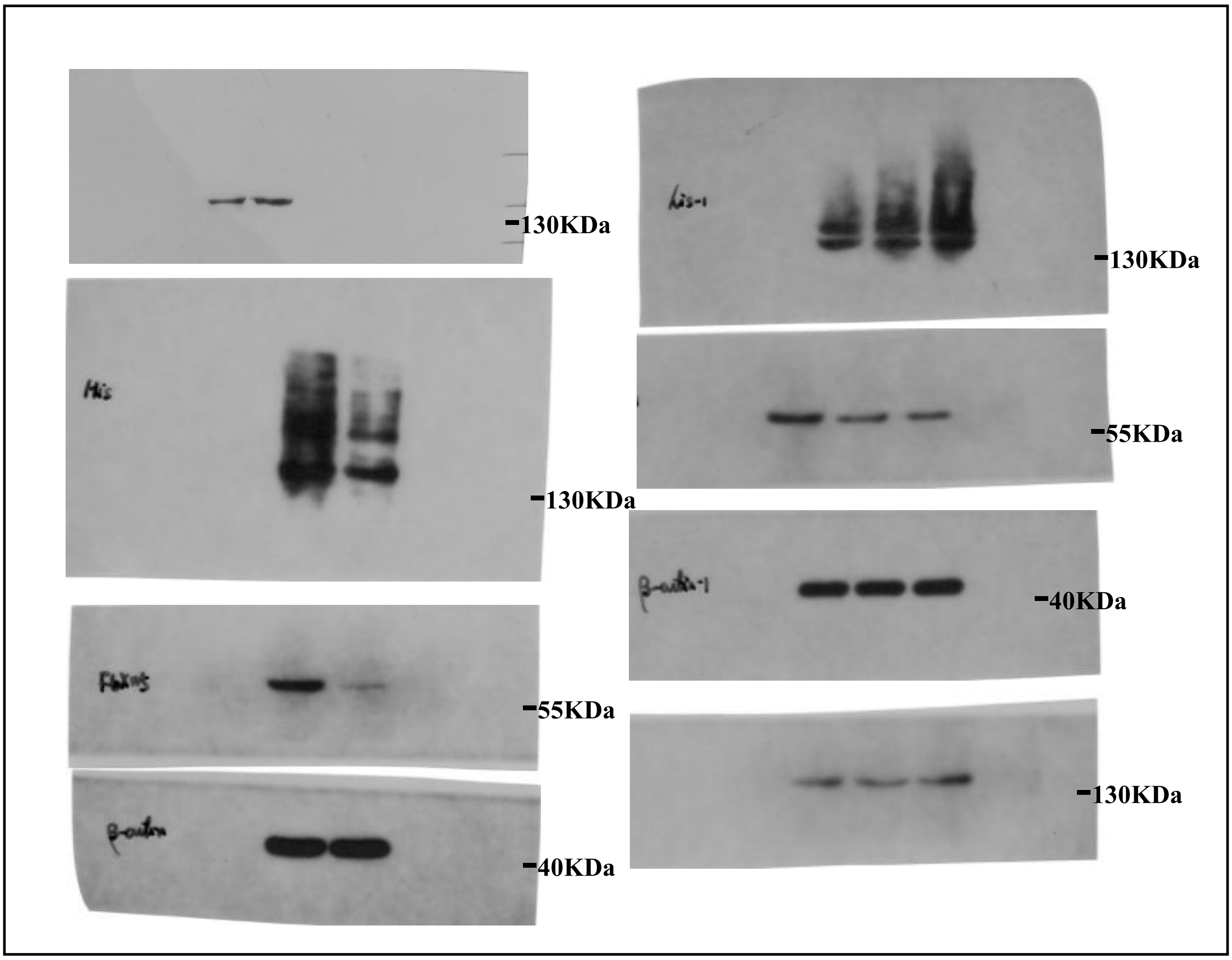

FIGURE 6A&E

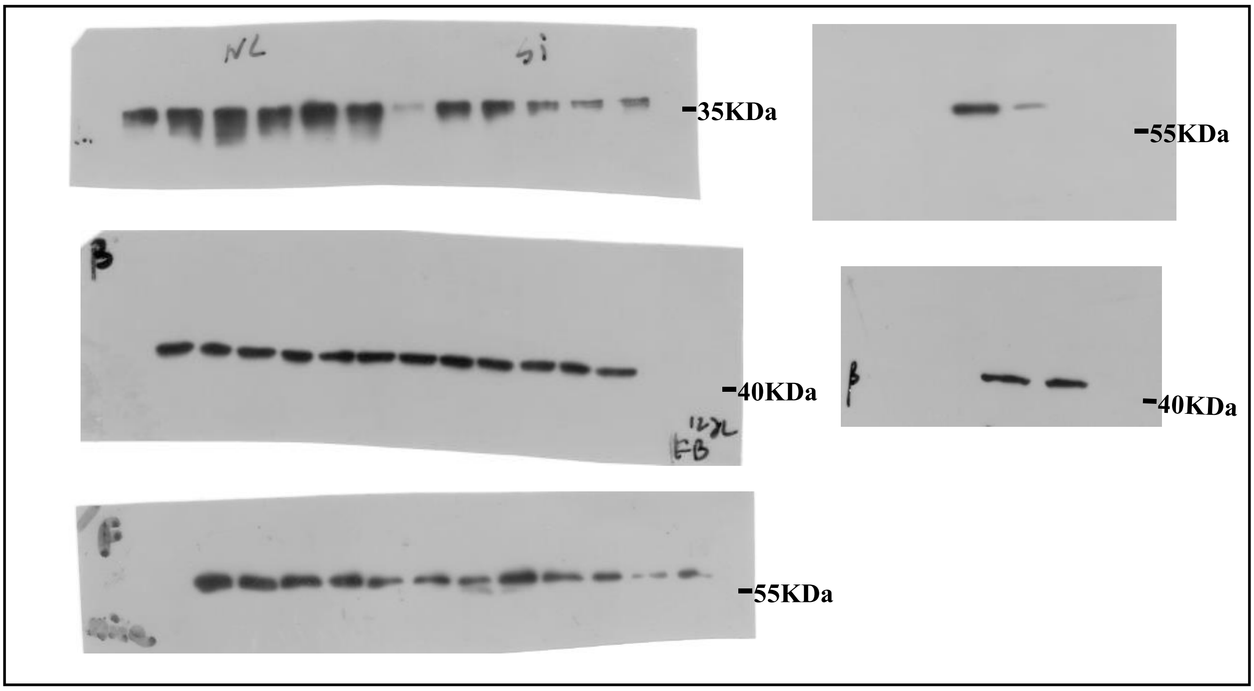

FIGURE 7A&C

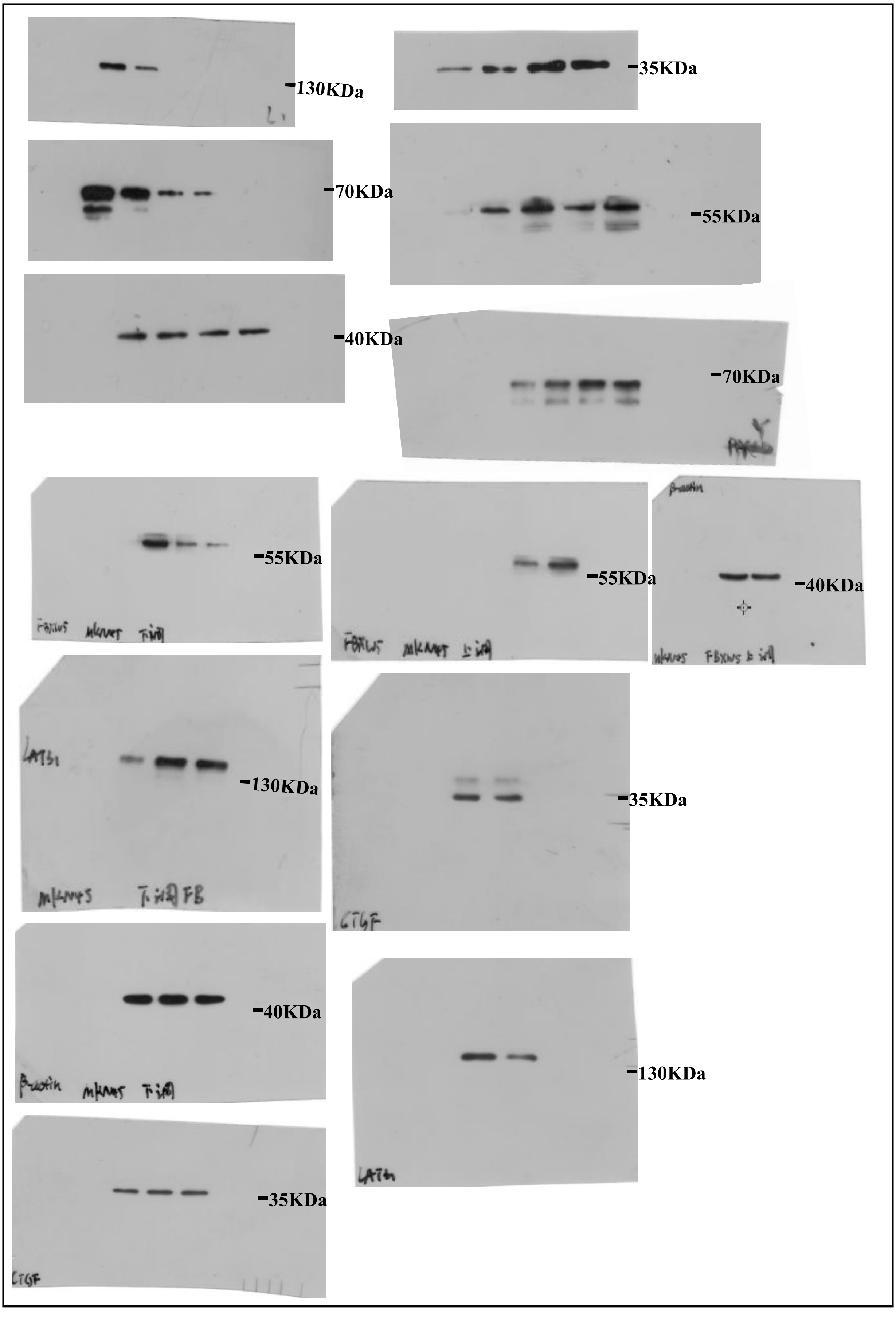

130KDa
35KDa
70KDa
55KDa
40KDa
70KDa
55KDa
55KDa
40KDa
130KDa
35KDa
40KDa
130KDa
35KDa
CTGF

FIGURE 7D-E

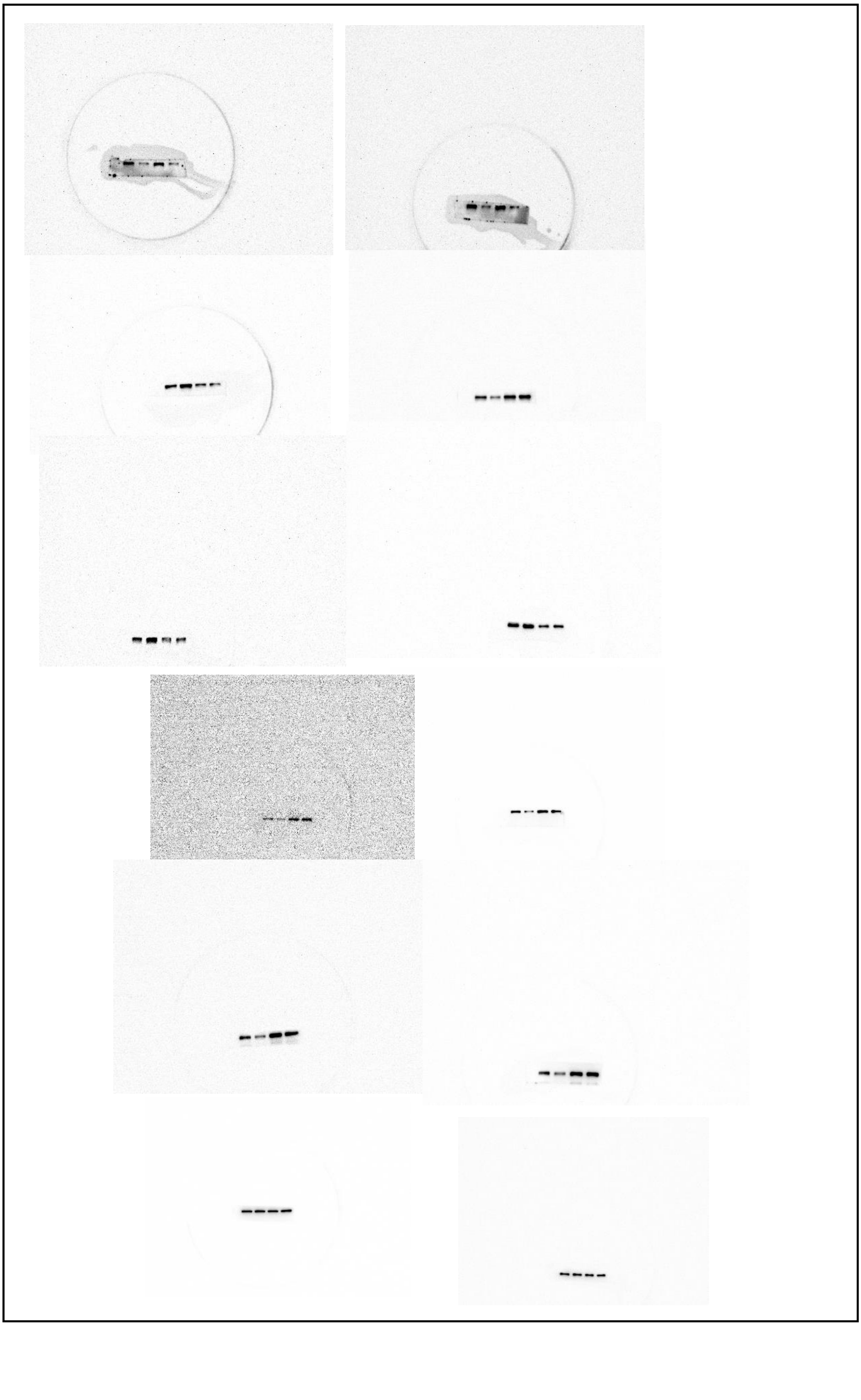

FIGURE S3A

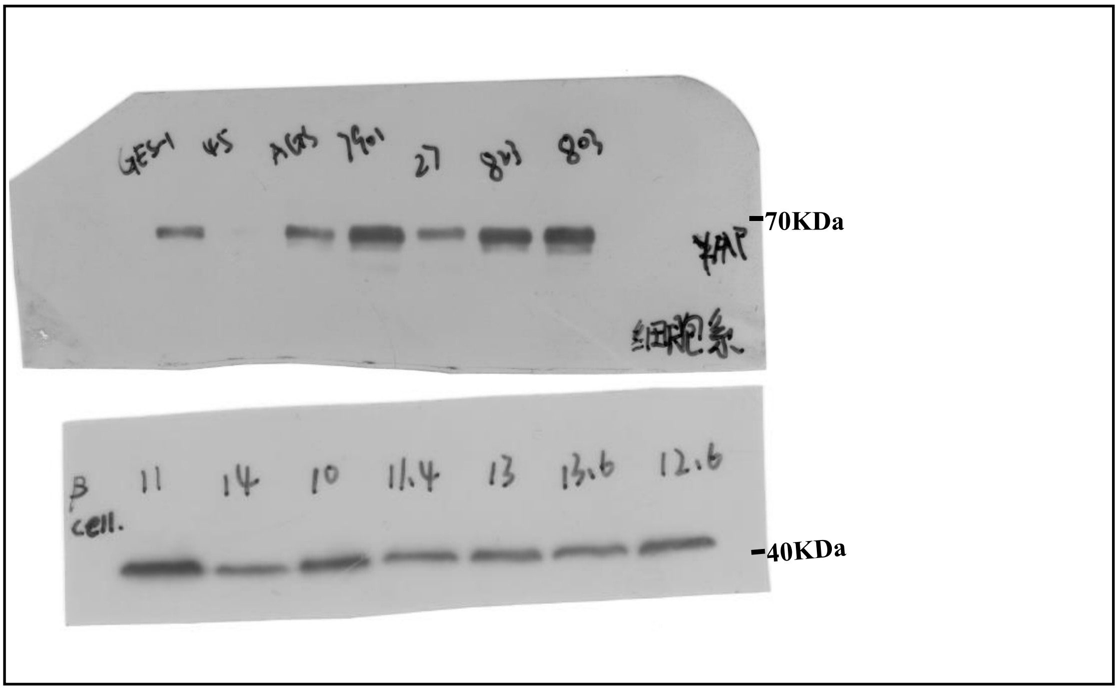

Supplement: Supplementary file 1 — western blots original data [file 41420_2022_868_MOESM1_ESM.pdf]
