# Supplementary material for: The E3 ubiquitin ligase, FBXW5, promotes the migration and invasion of gastric cancer through the dysregulation of the Hippo pathway
Source: Cell Death Discov. 2022 Feb 24;8:79. doi: 10.1038/s41420-022-00868-y (PMC8873275; doi:10.1038/s41420-022-00868-y)
Supplement: Supplementary file 2 — Supplementary figure and table legends [file 41420_2022_868_MOESM2_ESM.docx]

**Supplementary files**

| **Filename** | **Description** |
| --- | --- |
| **Figure S1** | **Supplementary Figure S1** The level of FBXW5 mRNA in human tumors and its significance in GC. (A) The mRNA expression of FBXW5 in various cancers based on the Human Protein Atlas database. (B) The Kaplan-Meier Plotter survival database predicted an association between high FBXW5 mRNA levels and poor survival in GC. |
| **Figure S2** | **Supplementary Figure S2** The function of FBXW5 and its regulation of the Hippo pathway. (A) Transwell assays were conducted after the upregulation or silencing of FBXW5 in the GC cells, AGS and MGC-803. (B) Quantitative real-time PCR was performed using MGC-803 cells to evaluate the effect of FBXW5 knockdown on the Hippo pathway target genes at the mRNA level. Bar scale: 100 μm. Data are presented as mean ± SEM from three biologically independent experiments. |
| **Figure S3** | **Supplementary Figure S3** Western blot analysis of YAP1 in the GC cell lines. |
| **Table S1** | **Supplementary Table S1** Primary antibodies used in this study |
| **Table S2** | **Supplementary Table S2** Sequences of the siRNAs used in this study |
| **Table S3** | **Supplementary Table S3** The primer sequences used for qPCR |
