# Supplementary material for: The E3 ubiquitin ligase, FBXW5, promotes the migration and invasion of gastric cancer through the dysregulation of the Hippo pathway
Source: Cell Death Discov. 2022 Feb 24;8:79. doi: 10.1038/s41420-022-00868-y (PMC8873275; doi:10.1038/s41420-022-00868-y)
Supplement: Supplementary file 3 — Supplementary Tables [file 41420_2022_868_MOESM3_ESM.docx]

Supplementary Table1 Primary antibodies used in this study

| Primary antibody name | Company | Cat No |
| --- | --- | --- |
| FBXW5 | Proteintech | 18321-1-AP |
| YAP1 | Cell Signaling Technology | 14074s |
| N-cadherin | Cell Signaling Technology | 13116 |
| vimentin | Cell Signaling Technology | 5741 |
| E-cadherin | Cell Signaling Technology | 14472 |
| Ki-67 | Servicebio | GB111141 |
| CD31 | Servicebio | GB11063-2 |
| p-YAP1 | Cell Signaling Technology | 13008 |
| LATS1 | Cell Signaling Technology | 3477 |
| LATS2 | Bethyl Laboratories | A300-479A |
| β-actin | Cell Signaling Technology | 4967 |
| MST1 | Cell Signaling Technology | 14946 |
| CTGF | Cell Signaling Technology | 86641 |

Supplementary Table2 Sequences of siRNAs involved in this study

| siRNA | Sequence | |
| --- | --- | --- |
| FBXW5 #1 | Sense: | 5′-CCCAGUUCUCCCAGUUCAATT -3′ |
|  | Antisense: | 5′-UUGAACUGGGAGAACUGGGTT -3′ |
| FBXW5 #2 | Sense: | 5′-GGCUGUUCAAGAUCCAGAATT-3′ |
|  | Antisense: | 5′-UUCUGGAUCUUGAACAGCCTT -3′ |
| LATS1 | Sense: | 5′-GGUAGUUCGUCUAUAUUAUTT-3′ |
|  | Antisense: | 5′-ACGUGACACGUUCGGAGAATT-3′ |
| Negative Control (NC) | Sense: | 5′-UUCUCCGAACGUGUCACGUTT-3′ |
|  | Antisense: | 5′-ACGUGACACGUUCGGAGAATT-3′ |

Supplementary Table3 The primer sequences used for qPCR

| Gene | Sequence | |
| --- | --- | --- |
| β-actin | Forward: | 5′-TCACCCACACTGTGCCCATCATCGA-3′ |
|  | Reverse: | 5′-CAGCGGAACCGCTCATTGCCAATGG-3′ |
| FBXW5 | Forward: | 5′- CCTCAGCTTCTCCCATTCCG -3′ |
|  | Reverse: | 5′- TGCTCCAGATCTTCACAGTGC -3' |
| CTGF | Forward: | 5′- GTTTGGCCCAGACCCAACTA -3′ |
|  | Reverse: | 5′- GGCTCTGCTTCTCTAGCCTG -3' |
| AREG | Forward: | 5′-TGTCGCTCTTGATACTCGGC-3′ |
|  | Reverse: | 5′-ATGGTTCACGCTTCCCAGAG-3′ |
| CYR61 | Forward: | 5′-CGCCTTGTGAAAGAAACCCG-3′ |
|  | Reverse: | 5′-GGTTCGGGGGATTTCTTGGT-3′ |
| CDX2 | Forward: | 5′-GAGTGGTGTACACGGACCAC-3′ |
|  | Reverse: | 5′-CTCCTTTGCTCTGCGGTTCT-3′ |
