# Supplementary figures and images for: The E3 ubiquitin ligase, FBXW5, promotes the migration and invasion of gastric cancer through the dysregulation of the Hippo pathway
Source: Cell Death Discov. 2022 Feb 24;8:79. doi: 10.1038/s41420-022-00868-y (PMC8873275; doi:10.1038/s41420-022-00868-y)

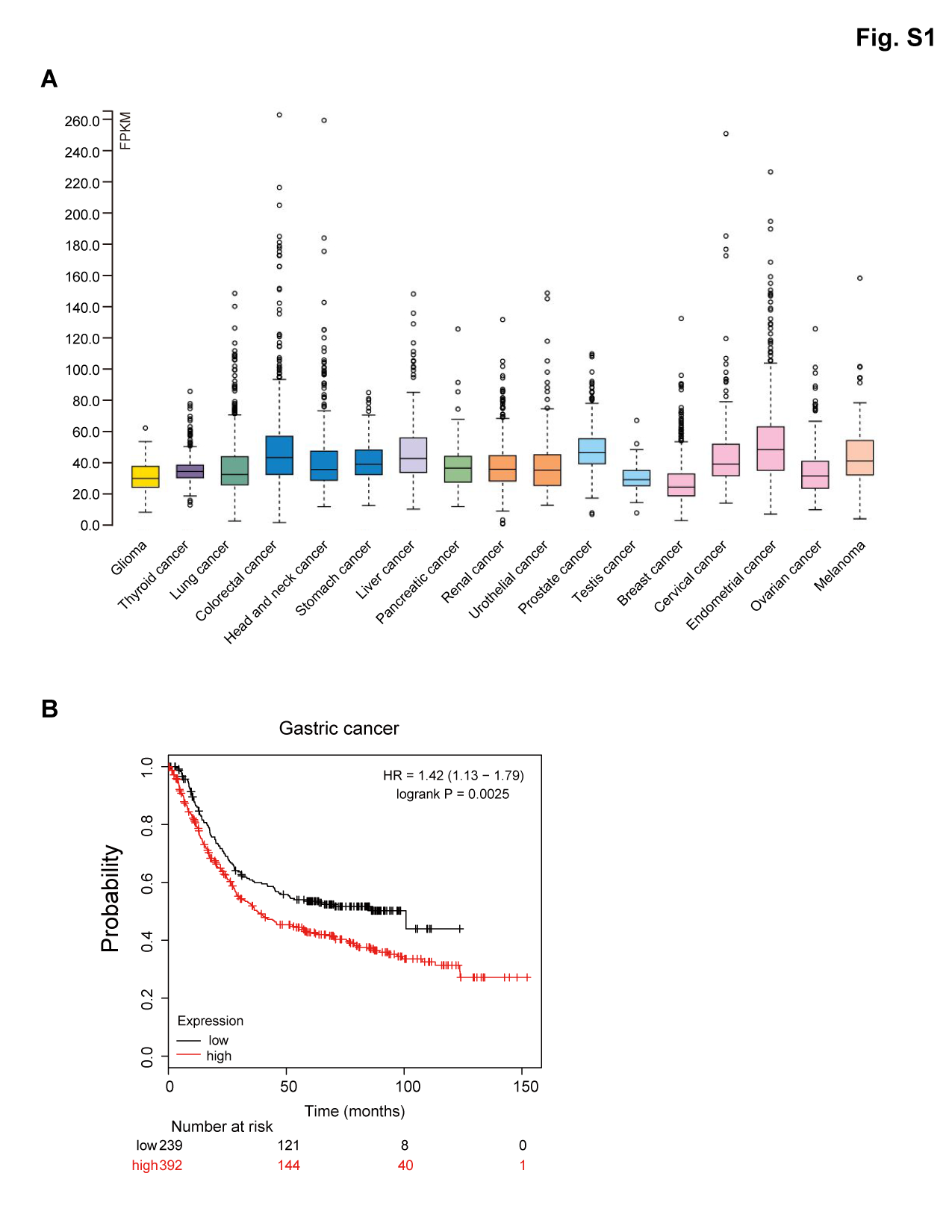

Supplement: Supplementary file 4 — Supplementary Figure S1 [file 41420_2022_868_MOESM4_ESM.tif]

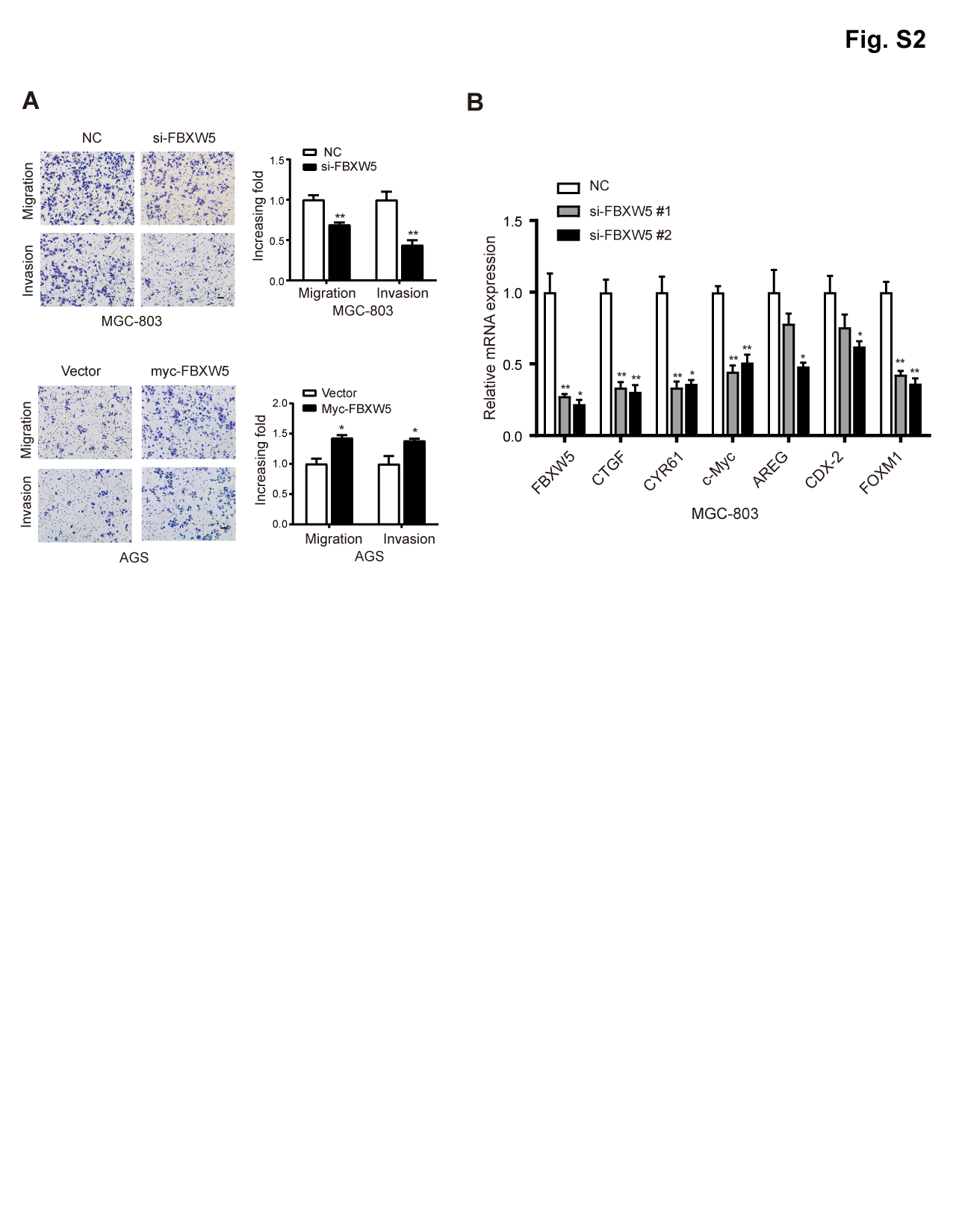

Supplement: Supplementary file 5 — Supplementary Figure S2 [file 41420_2022_868_MOESM5_ESM.tif]

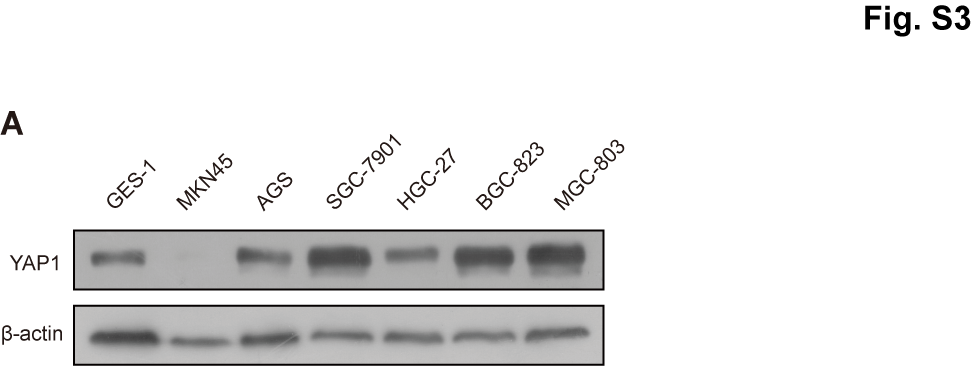

Supplement: Supplementary file 6 — Supplementary Figure S3 [file 41420_2022_868_MOESM6_ESM.tif]
